# Prevalencia de la resistencia a macrólidos y aminoglucósidos en los complejos *Mycobacterium avium* y *M. abscessus* y en *Mycobacterium chelonae* identificados en el Laboratorio Nacional de Referencia de Colombia entre el 2018 y el 2022

**DOI:** 10.7705/biomedica.7197

**Published:** 2024-05-30

**Authors:** Claudia Llerena, Yanely Angélica Valbuena, Angie Paola Zabaleta, Angélica Nathalia García

**Affiliations:** 1 Grupo de Micobacterias, Laboratorio Nacional de Referencia, Instituto Nacional de Salud, Bogotá, D.C., Colombia Laboratorio Nacional de Referencia Instituto Nacional de Salud Bogotá, D.C. Colombia

**Keywords:** micobacterias no tuberculosas, infecciones por *Mycobacterium*, *Mycobacterium avium*, *Mycobacterium chelonae*, *Mycobacterium abscessus*, macrólidos, aminoglucósidos, terapéutica, diagnóstico, Non-tuberculous mycobacteria, *Mycobacterium* infections, *Mycobacterium avium*, *Mycobacterium chelonae*, *Mycobacterium abscessus*, macrolides, aminoglycosides, therapeutics, diagnosis

## Abstract

**Introducción.:**

*Mycobacterium chelonae* y los complejos *Mycobacterium avium* y *M. abscessus*, son agentes patógenos emergentes causantes de micobacteriosis. El tratamiento de esta infección depende de la especie y la subespecie identificadas. Los fármacos de elección son los macrólidos y aminoglucósidos, contra los cuales se ha reportado resistencia; por esta razón, el determinar el perfil de sensibilidad le permite al médico tratante comprender mejor el pronóstico y la evolución de estas infecciones.

**Objetivo.:**

Describir los perfiles de sensibilidad ante macrólidos y aminoglucósidos, de los cultivos identificados como complejo *Mycobacterium avium,* complejo *M. abscessus* o especie *M. chelonae*, en el Laboratorio Nacional de Referencia de Micobacterias durante los años 2018 a 2022.

**Materiales y métodos.:**

Se llevó a cabo un estudio descriptivo del perfil de sensibilidad a macrólidos y aminoglucósidos, de los cultivos identificados como complejo *M. avium*, complejo *M. abscessus* o *M. chelonae*, mediante la metodología GenoType® NTM-DR.

**Resultados.:**

Los cultivos del complejo *M. avium* fueron 159 (47,3 %), de los cuales, 154 (96,9 %) fueron sensibles y 5 (3,1 %) resistentes a los macrólidos; todos fueron sensibles a los aminoglucósidos. Del complejo *M. abscessus* se estudiaron 125 (37,2 %) cultivos, 68 (54,4 %) resultaron sensibles y 57 (45,6 %) resistentes a los macrólidos; solo un cultivo (0,8 %) fue resistente a los aminoglucósidos. De *M. chelonae* se analizaron 52 cultivos (15,5 %), todos sensibles a los macrólidos y aminoglucósidos.

**Conclusiones.:**

En las tres especies de micobacterias estudiadas, la resistencia contra la amikacina fue la menos frecuente. La identificación de las subespecies y los perfiles de sensibilidad permiten instaurar esquemas de tratamiento adecuados, especialmente en las micobacteriosis causadas por *M. abscessus.*

## Abstract (en)

**Introduction.:**

The *Mycobacterium chelonae* species and the *M. avium* and *M. abscessus* complexes are emerging pathogens that cause mycobacteriosis. Treatment depends on the species and subspecies identified. The drugs of choice are macrolides and aminoglycosides. However, due to the resistance identified to these drugs, determining the microbe’s sensitivity profile will allow clinicians to improve the understanding of the prognosis and evolution of these pathologies.

**Objective.:**

To describe the macrolide and aminoglycoside susceptibility profile of cultures identified by Colombia’s *Laboratorio Nacional de Referencia de Mycobacteria* from 2018 to 2022, as *Mycobacterium avium* complex*, M. abscessus* complex, and *M. chelonae*

**Materials and methods.:**

This descriptive study exposes the susceptibility profile to macrolides and aminoglycosides of cultures identified as *M. avium* complex, *M. abscessus* complex, and *M. chelonae* using the GenoType® NTM-DR method.

**Results.:**

We identified 159 (47.3 %) cultures as *M. avium* complex, of which 154 (96.9 %) were sensitive to macrolides, and 5 (3.1 %) were resistant; all were sensitive to aminoglycosides. From the 125 (37.2 %) cultures identified as *M. abscessus* complex, 68 (54.4 %) were sensitive to macrolides, 57 (45.6 %) were resistant to aminoglycosides, and just one (0.8 %) showed resistance to aminoglycosides. The 52 cultures (15.5 %) identified as *M. chelonae* were sensitive to macrolides and aminoglycosides.

**Conclusions.:**

The three studied species of mycobacteria have the least resistance to Amikacin. Subspecies identification and their susceptibility profiles allow the establishment of appropriate treatment schemes, especially against *M. abscessus*.

La especie *Mycobacterium chelonae* y los complejos *Mycobacterium avium* y *Mycobacterium abscessus* son agentes patógenos emergentes presentes en el medio ambiente que causan enfermedades respiratorias o infecciones localizadas en piel, tejidos blandos, huesos y articulaciones, entre otras, las cuales pueden ser diseminadas en personas con inmunosupresión.

El complejo *M. avium* incluye las especies *M. avium*, *M. intracellulare*, *M. chimaera*, *M. colombiense*, *M. marseillense*, *M. timonense*, *M. boucherdurhonense*, *M. vulneris* y *M. arosiense*, clasificadas en el grupo III de Runyon como de crecimiento lento y no cromógenas. El complejo *M. abscessus* está compuesto por *M. abscessus* subsp*. abscessus*, *M. abscessus* subsp*. bolletii* y *M. abscessus* subsp. *masiliense*, y junto con *M. chelonae*, están clasificadas en el grupo IV de Runyon como especies de crecimiento rápido ^1^^-^^3^.

El tratamiento de estas infecciones depende de la especie identificada y la correlación de estos hallazgos con las manifestaciones clínicas. Los fármacos de elección son los macrólidos, cuyo efecto es bacteriostático, aunque en dosis elevadas, pueden comportarse como bactericidas. La claritromicina es uno de los más efectivos contra el complejo *M. avium* y *M. chelonae.* Los aminoglucósidos como la amikacina también son utilizados porque tienen actividad bactericida ^4^.

Los complejos *M. avium* y *M. abscessus* junto con *M. chelonae* son microrganismos que han desarrollado una resistencia natural o adquirida a los fármacos. La primera se debe a que las micobacterias no tuberculosas poseen características especiales para tolerarlos y la segunda es consecuencia de una exposición no controlada que ocasiona falla terapéutica. Por tal razón, cuando una persona se somete a estos tratamientos, es necesario combinar más de dos medicamentos, cuyo tiempo de administración depende de la evolución clínica y bacteriológica; esta última se evalúa mediante cultivo: si es negativo por más de seis meses, la persona se considera curada. ^5^^-^^8^.

Es muy relevante tener la identificación correcta de la especie. Por ejemplo, en una revisión sistemática de Diel *et al*., se documentó una tasa de mortalidad superior al 25 % en cinco años por especies del complejo *M. avium*. Se conoce que especies como *M. abscessus* y *M. chelonae* tienen similitudes genéticas que dificultan su identificación mediante pruebas de laboratorio y que pueden llevar a suministrar un tratamiento incorrecto, pues *M. abscessus* presenta altas tasas de resistencia a los fármacos: las subespecies *abscessus* y *bolletii* tienen resistencia intrínseca a los macrólidos. Aunque son pocos los métodos de laboratorio que proporcionan esta información, es fundamental fortalecer la vigilancia de la resistencia a fármacos en estas micobacterias ^8^^-^^10^.

El presente estudio tiene como objetivo describir las especies, las subespecies y los respectivos perfiles de sensibilidad a macrólidos y aminoglucósidos de los cultivos identificados como complejo *M. avium*, complejo *M. abscessus* o *M. chelonae*, en el Laboratorio Nacional de Referencia de Micobacterias del Instituto Nacional de Salud de Colombia, durante los años 2018 a 2022, utilizando la metodología GenoType® NTM-DR.

## Materiales y métodos

Se llevó a cabo un estudio descriptivo con los cultivos identificados como complejo *M. avium*, complejo *M. abscessus* o *M. chelonae* en el Instituto Nacional de Salud de Colombia en el marco del proceso de vigilancia de micobacterias no tuberculosas que adelantó el Laboratorio Nacional de Referencia de Micobacterias durante los años 2018 a 2022.

Los cultivos crioconservados se recuperaron en medio de cultivo líquido MGIT^TM^ (*Mycobacteria Growth Indicator Tube*) que contiene caldo Middlebbrok 7H9 y suplementos para potenciar el crecimiento. El proceso de incubación se hizo en el equipo Bactec^TM^ MGIT^TM^. Se seleccionaron los cultivos previamente identificados como complejo *M. avium*, complejo *M. abscessus* o *M. chelonae.* No se excluyó ningún cultivo del periodo seleccionado.

Cuando se observó crecimiento en el medio de cultivo, se procedió con la extracción de ADN, empleando el kit de Genolyse®. Se hizo la prueba para detectar genes relacionados con la resistencia a los macrólidos y aminoglucósidos mediante el kit GenoType® NTM-DR V. 1.0, siguiendo las instrucciones del fabricante. Esta técnica se fundamenta en la amplificación del ADN de interés, mediante la reacción en cadena de la polimerasa, y en la visualización de los productos de amplificación, mediante hibridación en tiras de nitrocelulosa. La técnica permite identificar las subespecies del complejo *M. avium*, que son: *M. avium*, *M. intracellulare* y *M. chimaera*; en el complejo *M. abscessus*, las subespecies son *abscessus*, *bolletii* y *masiliense*; y, finalmente, la especie *M. chelonae.* Además, permite evaluar el gen *erm* para establecer la sensibilidad a los macrólidos en los miembros del complejo *M. abscessus* y, el gen *rrl*, para las demás especies. Para identificar la resistencia a los aminoglucósidos, se usa el gen *rrs.*^11^.

Como control interno, se usó la cepa ATCC® 15769^TM^, correspondiente a *M. avium* subsp. *M. avium*. Las etapas de extracción, amplificación e hibridación se ejecutaron según los métodos del Laboratorio Nacional de Referencia de Micobacterias.

Las fuentes de información fueron el formato único de vigilancia de micobacterias y la base de datos del biobanco del Laboratorio Nacional de Referencia de Micobacterias del Instituto. Las variables analizadas fueron: especie o subespecie identificadas, tipo de muestra sembrada (pulmonar, cuando era esputo, lavado bronquial o broncoalveolar, aspirado o lavado gástrico, o biopsia de pulmón; piel para muestras de biopsia o secreción de piel; y muestras extrapulmonares que incluían biopsias o secreciones de órganos, líquidos, sangre y materia fecal), y el perfil de sensibilidad a los macrólidos (claritromicina, azitromicina) y los aminoglucósidos (kanamicina, amikacina y gentamicina).

## Resultados

Durante los años 2018 a 2022, se recibieron 336 aislamientos. De estos, 159 (47,3 %) correspondían al complejo *M. avium*, 125 (37,2 %) al complejo *M. abscessus* y 52 (15,5 %) a *M. chelonae*.

Se identificó el complejo *M. avium* en 125 (78,6 %) aislamientos de muestras pulmonares, en 28 (17,6 %) extrapulmonares y en 6 (3,8 %) muestras de piel. Del total, 154 (96,8 %; IC_95%_: 92,8-98,9) fueron sensibles a macrólidos y 5 (3,1 %; IC_95%_: 1,0-7,2) fueron resistentes; todos estos aislamientos fueron sensibles a los aminoglucósidos (100 %; IC_95%_: 97,7-100).

Desagregando por especies del complejo *M. avium,* se encontró *M. intracellulare* en 84 (83,2 %) aislamientos de origen pulmonar, 15 (14,9 %) en muestras extrapulmonares y 2 (2 %) en piel; 98 cepas (97 %; IC_95%_: 91,6 99,4) fueron sensibles a los macrólidos y, 101 (100 %; IC_95%_: 96,4-100), a los aminoglucósidos. *Mycobacterium avium* se identificó en 25 (61 %) cultivos de muestras pulmonares, en 12 (29,3 %) extrapulmonares y en 4 (9,8 %) de piel; 39 cultivos (95,1 %; IC_95%_: 83,5-99,4) fueron sensibles a los macrólidos y 41 (100 %; IC_95%_: 91,4-100) a los aminoglucósidos. *Mycobacterium chimaera* se identificó en 16 (94 %) aislamientos de origen pulmonar y en una (6 %) muestra extrapulmonar; todos fueron sensibles a los fármacos evaluados (100 %; IC_95%_: 91,4-100) (cuadro 1).

**Cuadro 1.** Casos de micobacteriosis causados por el complejo *Mycobacterium avium* y su perfil de sensibilidad (Colombia, 2018-2022)

**Complejo *Mycobacterium avium***					
Especie identificada y tipo de muestra		Macrólidos		Aminoglucósidos	
		(claritromicina, azitromicina)		(kanamicina, amikacina, gentamicina)	
*M. intracellulare*		Sensible	Resistente	Sensible	Resistente
	n (%)	n (%)	n (%)	n (%)	n (%)
Pulmonar	84 (83,2)	81 (96,4)	3 (3,6)	84 (100)	0 (0)
Extrapulmonar	15 (14,9)	15 (100)	0 (0)	15 (100)	0 (0)
Piel	2 (2,0)	2 (100)	0 (0)	2 (100)	0 (0)
Subtotal	101 (100,0)	98 (97)	3 (3)	101 (100)	0 (0)
		IC_95%_: 91,6-99,4	IC_95%_: 0,6 -8,4	IC_95%_: 96,4-100	IC_95%_: 0,0-3,6
*M. avium*		Sensible	Resistente	Sensible	Resistente
	n (%)	n (%)	n (%)	n (%)	n (%)
Pulmonar	25 (61)	23 (92)	2 (8)	25 (100)	0 (0)
Extrapulmonar	12 (29,3)	12 (100)	0 (0)	12 (100)	0 (0)
Piel	4 (9,8)	4 (100)	0 (0)	4 (100)	0 (0)
Subtotal	41 (100)	39 (95,1)	2 (4,9)	41 (100)	0 (0)
		IC_95%_: 83,5-99,4	IC_95%_: 0,6-16,5	IC_95%_: 91,4-100	IC_95%_: 0,0-8,6
*M. chimaera*		Sensible	Resistente	Sensible	Resistente
	n (%)	n (%)	n (%)	n (%)	n (%)
Pulmonar	16 (94)	16 (100)	0 (0)	16 (100)	0 (0)
Extrapulmonar	1 (6)	1 (100)	0 (0)	1 (100)	0 (0)
Subtotal	17 (100)	17 (100)	0 (0)	17 (100)	0 (0)
		IC_95%_: 80,5-100	IC_95%_: 0,0-19,5	IC_95%_: 80,5-100	IC_95%_: 0,0-19,5
Total	159 (100)	154 (96,8)	5 (3,1)	159 (100)	0 (0)
		IC_95%_: 92,8-98,9	IC_95%_: 1,0-7,2	IC_95%_: 97,7-100	IC_95%_: 0,0-2,3

IC: intervalo de confianza

El complejo *M. abscessus* se aisló en 90 (72 %) muestras pulmonares, en 29 (23,2 %) de piel, en 4 (3,2 %) de líquido cefalorraquídeo y biopsias extrapulmonares, y en 2 (1,6 %) que no tenían dato de origen; 68 (54,4 %; IC_95%_: 45,3-63,5) aislamientos fueron sensibles a los macrólidos y 124 (99,2 %; IC_95%_: 95,6-99,9) a los aminoglucósidos.

Discriminando por subespecies, se aisló *M. abscessus* en 50 (79,4 %) muestras pulmonares, en 12 (19 %) de piel y en una (1,6 %) de médula ósea; 19 (30,2 %; IC_95%_: 18,0-42,3) fueron sensibles a los macrólidos y 63 (100 %; IC_95%_: 94,3-100) a los aminoglucósidos. La subespecie *massiliense* se identificó en 30 (62,5 %) cultivos de muestras pulmonares, en 15 (31,3 %) de piel, en 2 (4,2 %) de líquido cefalorraquídeo y en una (2,1 %) que no tenía dato de origen; todas (100 %; IC_95%_: 92,6-100) fueron sensibles a los macrólidos y 48 (100%; IC _95%_ 92,6 - 100) a los aminoglucósidos. En la subespecie *bolletii,* 10 (71,4 %) aislamientos fueron de origen pulmonar, 2 (14,3 %) de piel, uno (7,1 %) de secreción ganglionar y uno (7,1 %) no tenía el dato; solo un cultivo (7,1 %; IC_95%_: 0,2-33,9) fue sensible a los macrólidos y 14 (100 %; IC_95%_: 76,8-100) a los aminoglucósidos (cuadro 2).

**Cuadro 2:** Casos de micobacteriosis causados por el complejo *Mycobacterium abscessus* y su perfil de sensibilidad (Colombia, 2018-2022)

**Complejo *Mycobacterium abscessus***					
Especie identificada y tipo de muestra		Macrólidos (claritromicina, azitromicina)		Aminoglucósidos (kanamicina, amikacina, gentamicina)	
**Subsp. *M. abscessus***		Sensible	Resistente	Sensible	Resistente
	n (%)	n (%)	n (%)	**n (%)**	n (%)
Pulmonar	50 (79,4)	15 (30)	35 (70)	50 (100)	0 (0)
Extrapulmonar	12 (19)	4 (33,3)	8 (66,7)	12 (100)	0 (0)
Piel	1 (1,6)	0 (0)	1 (100)	1 (100)	0 (0)
Subtotal	63 (100)	19 (30,2) IC_95%_: 18,0-42,3	44 (69,8) IC_95%_: 57,7-81,9	63 (100) IC_95%_: 94,3-100	0 (0) IC_95%_: 0,0-5,7
**Subsp. *M. massiliense***		Sensible	Resistente	Sensible	Resistente
	n (%)	n (%)	n (%)	n (%)	n (%)
Pulmonar	30 (62,5)	30 (100)	0 (0)	29 (96,7)	1 (3,3)
Piel	15 (31,3)	15 (100)	0 (0)	15 (100)	0 (0)
Extrapulmonar	2 (4,2)	2 (100)	0 (0)	2 (100)	0 (0)
Sin dato	1 (2,1)	1 (100)	0 (0)	1 (100)	0 (0)
Subtotal	48 (100)	48 (100) IC_95%_: 92,6-100	0 (0) IC_95%_: 0,0-7,4	47 (97,9) IC_95%_: 88,9-99,9	1 (2,1) IC_95%_: 0,05-11,1
**Subsp. *M. bolletii***		Sensible	Resistente	Sensible	Resistente
	n (%)	n (%)	n (%)	n (%)	n (%)
Pulmonar	10 (71,4)	1 (10)	9 (90)	10 (100)	0 (0)
Piel	2 (14,3)	0 (0)	2 (100)	2 (100)	0 (0)
Extrapulmonar	1 (7,1)	0 (0)	1 (100)	1 (100)	0 (0)
Sin dato	1 (7,1)	0 (0)	1 (100)	1 (100)	0 (0)
Subtotal	14 (100)	1 (7,1) IC_95%_: 0,2-33,9	13 (92,8) IC_95%_: 66,1-99,8	14 (100) IC_95%_: 76,8-100	0 (0) IC_95%_: 0,0-23,2
Total	125 (100)	68 (54,4) IC_95%:_ 45,3-63,5	57 (45,6) IC_95%_: 36,5-54,7	124 (99,2) IC_95%_: 95,6-99,9	1 (0,8) IC_95%_: 0,02-4,4

IC: intervalo de confianza

Se aisló *M. chelonae* en 45 (86,6 %) muestras pulmonares, en 5 (9,6 %) de piel, en una (1,9 %) de tejido ocular y en una (1,9 %) sin el dato; todos los aislamientos fueron sensibles a los macrólidos y los aminoglucósidos (100%; IC_95%_: 93,1-100) (cuadro 3).

**Cuadro 3:** Casos de micobacteriosis causados por *M. chelonae* y su perfil de sensibilidad (Colombia, 2018-2022)

*Mycobacterium chelonae*						
Especie identificada y tipo de muestra			Macrólidos (claritromicina, azitromicina)		Aminoglucósidos (kanamicina, amikacina, gentamicina)	
*M. chelonae*			Sensible	Resistente	Sensible	Resistente
	n	(%)	n (%)	n (%)	n (%)	n (%)
Pulmonar	45	(86,6)	45 (100)	0 (0)	45 (100)	0 (0)
Piel	5	(9,6)	5 (100)	0 (0)	5 (100)	0 (0)
Extrapulmonar	1	(1,9)	1 (100)	0 (0)	1 (100)	0 (0)
Sin dato	1	(1,9)	1 (100)	0 (0)	1 (100)	0 (0)
Total	52 (100)		52 (100)	0 (0)	52 (100)	0 (0)
			IC_95%_: 93,1-100	IC_95%_: 0,0-6,8	IC_95%_: 93,1-100	IC_95%_: 0,0-6,8

IC: intervalo de confianza

## Discusión

Los estudios realizados en diversos países demuestran que las especies de *Mycobacterium* no tuberculosas más prevalentes son las pertenecientes al complejo *M. avium*, seguidas de las del complejo *M. abscessus*, igual a lo observado en este trabajo ^12^^-^^14^.

En 2021, Mora *et al*. describieron el comportamiento epidemiológico y clínico de las micobacteriosis en Latinoamérica, y reportaron a *M. avium* como la causa más frecuente ^15^. Este hallazgo es igual a lo descrito en 2019 por Maurer *et al*., quienes identificaron a *M. intracellulare* y *M. avium* como las micobacterias más comunes, similar a lo aquí descrito. Informaron una resistencia a la claritromicina de 4,63 % y a la amikacina de 10,19 %, sin diferencias significativas entre las especies *M. intracellulare* y *M. avium*^16^.

En una revisión sistemática publicada en 2021, se registró resistencia a la claritromicina del 9,0 % (IC_95%_: 3,0-17,0%), en el grupo de aislamientos clínicos del complejo *M. avium*^17^. Wetzstein *et al*., en una cohorte de 85 pacientes, identificaron una resistencia a los macrólidos del 1,2 % (IC_95%_: 0,7-7,3) y destacaron que este hallazgo se relacionaba con la positividad de los cultivos realizados durante el seguimiento de los casos. Sin embargo, en este trabajo no se encontró resistencia a los aminoglucósidos ^18^. En 2018, Litvinov *et al*. evidenciaron resistencia a los macrólidos del 4,3 % y a la amikacina del 9,3 %, en una muestra de 363 cultivos ^19^. En el Hospital de Pulmón de Shanghái, de enero de 2019 a mayo de 2020, se obtuvieron 45 aislamientos de *M. avium* y 242 de *M. intracellulare*, cuya sensibilidad a la claritromicina fue de 88,89 % y 91,32 %, respectivamente, sin diferencias significativas entre las especies (p=0,601) ^14^.

Los datos de este estudio para Colombia son similares a los reportados por otros autores y se destaca que todos los cultivos del complejo *M. avium* fueron sensibles a la amikacina, igual a lo descrito por Wetzstein *et al*. ^18^.

Previamente, se ha reportado que el mayor porcentaje de micobacteriosis pulmonares son causadas por el complejo *M. abscessus*, el cual se considera de importancia en salud pública por ser las bacterias de este complejo un grupo que presenta gran resistencia a los antibióticos. Recientemente, se describió su trasmisión de persona a persona en individuos con fibrosis quística, aumentando su morbilidad y mortalidad. La subespecie más aislada es *M. abscessus*, seguida de *M. massiliense* y, en baja proporción, *M. bolletii*, similar a los datos encontrados en el presente estudio ^20^^-^^25^.

En 2020, Weng *et al.* reportaron que en Taiwán, la sensibilidad a los macrólidos era del 53 al 93 % a la claritromicina, del 52 % a la azitromicina y del 93 al 96 % a la amikacina; en Japón, se encontró una sensibilidad del 62 al 69 %, en Corea del Sur, del 78 %, en Tailandia, del 52 al 92 %, y en China, del 99 al 100 %; en Europa fue del 49 al 85 % y algunos países de América tienen una sensibilidad del 85 % ^23^. En Venezuela, en un estudio realizado en cultivos obtenidos de 2004 a 2009, se encontró por subespecies una sensibilidad a amikacina del 100 %, una resistencia a ciprofloxacina del 86 % en *M. abscessus,* del 66 % en *M. massiliense* y del 100 % en *M. bolletii*^26^. En el 2021, en China se reportó resistencia inducida a la claritromicina del 65 % en *M. abscessus* y del 2 % en *M. massiliense;* y una resistencia adquirida del 17 % para *M. abscessus* y del 8 % para *M. massiliense*; para la amikacina la sensibilidad fue del 94 % ^25^.

En un estudio realizado en Shanghái, con cultivos obtenidos del 2014 al 2018, encontraron una sensibilidad a la amikacina del 96,9 % para *M. abscessus* y del 100,0 % para *M. massiliense*; la resistencia a claritromicina fue de 68,8 % para *M. abscessus* y más del 80 % de las cepas de la subespecie *M. massiliense* fueron sensibles. En este estudio no se identificó *M. bolletii*^24^.

En general, los datos mencionados muestran una sensibilidad mayor del 90 % del complejo *M. abscessus* a la amikacina. En el caso de la claritromicina, se observan variaciones que pueden estar relacionadas con la diferencia en el número de cultivos estudiados en cada uno de estos trabajos y la epidemiología de las micobacteriosis en cada región o país. Sin embargo, se resalta que la gran resistencia de las subespecies *M. abscessus*, *M. bolletii* y *M. massiliense* a este fármaco, se debe a que tienen el gen *erm* no funcional, lo que fundamenta la necesidad de contar con pruebas que permitan una identificación precisa de la especie y la subespecie, ya que la conducta terapéutica es diferente. Esto también se relaciona con la tasa de éxito del tratamiento, que es mayor en pacientes con *M. massiliense* en comparación con los infectados con *M. abscessus*^20^^,^^23^^,^^24^.

*Mycobacterium chelonae* causa infecciones intrahospitalarias y afecta los tejidos blandos (piel) y los ojos; también, puede ocasionar enfermedad pulmonar o diseminada e infecciones invasivas asociadas con el uso de catéteres. En este estudio, el mayor número de aislamientos provenía de muestras pulmonares, seguidos por aquellos de piel; este hallazgo se relaciona con factores de riesgo predisponentes, como la enfermedad pulmonar obstructiva crónica o el antecedente de tratamiento para tuberculosis, que hacen a estas personas más susceptibles de adquirir micobacterias no tuberculosas. En cuanto a la sensibilidad a los medicamentos, en un estudio de 26 cultivos de *M. chelonae* realizado en Turquía, se identificó una sensibilidad a claritromicina del 92 %, mientras que, en el presente trabajo, todos los cultivos fueron sensibles a los macrólidos y aminoglucósidos. Esto permite considerar que la resistencia a fármacos en *M. chelonae* aún no es un problema; sin embargo, se debe mantener la vigilancia ^27^^-^^29^.

Los resultados obtenidos coinciden con los trabajos publicados que mencionan a la amikacina como el medicamento más efectivo para el tratamiento de las tres especies estudiadas de *Mycobacterium*. Se resalta la importancia de identificar las subespecies y sus perfiles de sensibilidad, para determinar el manejo clínico y establecer un esquema de tratamiento adecuado, especialmente, en las infecciones causadas por *M. abscessus*^12^^,^^23^^,^^24^.
